# A Comparative Study of Isolated Secondary Metabolites from Lichens and Their Antioxidative Properties

**DOI:** 10.3390/plants11081077

**Published:** 2022-04-15

**Authors:** Ján Elečko, Mária Vilková, Richard Frenák, Deepti Routray, Dajana Ručová, Martin Bačkor, Michal Goga

**Affiliations:** 1 NMR Laboratory, Department of Chemistry, Faculty of Science, Pavol Jozef Šafárik University, Moyzesova 11, 040 01 Košice, Slovakia; jan.elecko@upjs.sk (J.E.); maria.vilkova@upjs.sk (M.V.); 2 Department of Botany, Institute of Biology and Ecology, Faculty of Science, Pavol Jozef Šafárik University, Mánesova 23, 041 67 Košice, Slovakia; richard.frenak@student.upjs.sk (R.F.); deepti.routray@upjs.sk (D.R.); dajana.rucova@upjs.sk (D.R.); martin.backor@upjs.sk (M.B.); 3 Institute of Biotechnology, Faculty of Biotechnology and Food Sciences, Slovak University of Agriculture in Nitra, 949 76 Nitra, Slovakia

**Keywords:** superoxide anion, DPPH, antioxidant activity, secondary metabolites, lichens, NMR

## Abstract

Free radicals play a critical role in the chemical processes that occur in all cells. Pharmaceutical companies manufacture a variety of synthetically prepared antioxidants, but it is known that many of these can be carcinogenic. As a result, efforts are being made to find natural antioxidants that do not have these side effects. Lichens may be suitable candidates because they contain secondary metabolites with proven antioxidant properties. This could be explained by the presence of compounds with phenolic groups in lichens. The radical scavenging reaction is a chemical reaction governed by stoichiometry, and our aim is to determine the efficacy of these reactions. The aim of this study is to compare metabolite activity based on the same amount of substance involved in radical scavenging, calculated in micromoles rather than weight concentration. This provides an accurate way of comparing radical scavenging activity. We tested superoxide anion scavenging activity and free radical scavenging activity of isolated lichen secondary metabolites and their mixtures in different ratios. The following compounds were isolated and tested for antioxidant activity: gyrophoric acid (*Umbilicaria hirsuta*), evernic acid (*Evernia prunastri*), physodic acid, 3-hydroxyphysodic acid, physodalic acid and atranorin (*Hypogymnia physodes*), and usnic acid (as a synthetic compound). Of all the tested compounds, 3-hydroxyphysodic acid, as well as mixtures containing this metabolite, showed the strongest scavenging activity. The results also demonstrated that calculation by amount of substance leads to a new consideration of antioxidant activity.

## 1. Introduction

Lichens belong to a distinctive group of organisms that base their life strategy upon symbiosis. Fungi (mycobiont) and green algae or cyanobacteria (photobionts) engage in this mutually beneficial relationship [1]. In addition, associated bacteria [2], yeasts [3], and other fungi [4] are present to make up variable complex interactions in lichens. This symbiotic way of life resulted in interesting new growth forms of lichens that look unlike any other closely related organisms and their known ancestors or even their isolated bionts [5].

These slow-growing, inconspicuous organisms, which were neglected by the scientific community for a long time, have been given their due in recent years by many researchers for their secondary metabolism, which contains numerous substances unique to them [6]. This is primarily because of various biological activities that these substances seem to exhibit, such as antimicrobial, antifungal, antiviral, antitumour, antipyretic, analgesic, anti-inflammatory, antigenotoxic, antioxidant, herbicidal, and insecticidal activities [7].

Antioxidants are substances that are very important in protection against oxidative processes. There are many resources of antioxidants in the plant kingdom as well as in fungi. These bioactive substances, which belong to phenols, flavonoids, and also carotenoids, play an important role in the prevention of chronic diseases, cancer, heart disease, or neurodegenerative diseases [8]. Antioxidants can be either natural or synthetically prepared [7]. Lichens are a rich source of natural antioxidants. Mostly phenolic compounds present a major group of secondary metabolites and play an important role in the regulation of lichen growth, as well as development and protection. It is known that lichens belong to the cryptogams, which settle in harsh environments with unpropitious climate conditions [7]. It is not surprising that there is a high chance of oxidative stress, and synthesis of these compounds increases their antioxidant capacities [9].

Antioxidant activity is one of the characteristic bioactivities of lichens mainly due to secondary compounds with phenolic units. The most commonly used antioxidants are, for example, butylated hydroxyanisole (BHA), butylated hydroxytoluene (BHT), tert-butylhydroquinone (TBHQ), and propyl gallate (PG), which are all synthetically made. Low cost and high efficiency are connected to synthetic antioxidants, but, considering their carcinogenic potential, natural replacement is still required [10].

Lichen extracts include a wide range of antioxidants, which was confirmed in many studies [11,12,13,14]. In all of these studies, free radical scavenging by DPPH was considered, and, in a few of them, the reduction in power or superoxide anion scavenging activity were also tested.

Another possible application of lichen secondary metabolites concerning antioxidant activity is in their therapeutic potential. Many diseases are accompanied by oxidative stress, and it plays a fundamental role in the pathophysiology of neurodegenerative diseases [8]. Thus far, human clinical trials were not successful; therefore, further extensive research is needed due to the complex nature of these diseases. Nonetheless, one of the potential reasons for these unsatisfying results is the question of antioxidant mechanisms and the possibility of a need for a variety of antioxidants to work together in order to have an effect [15]. Thus, the novelty of new natural compounds with antioxidant activity is necessary.

Radical scavenging is a chemical reaction that is governed by a certain stoichiometry, and our aim is to determine the effectivity of these reactions. Therefore, we chose the same amount of substance for all lichen metabolites, which should be equal to the amount of radical with which they react. Many other studies about antioxidant screening used the same weights to compare the effectivity of the substances. We suppose that calculation by the amount of substance leads to a new consideration of antioxidant activity. All substances have different molecular weights, which means that a different number of molecules participate in the reaction. Using the calculation of substance, we compared how the same number of molecules of a given substance is characterized by the ability of free radical scavenging, instead of the same weight of substances. The fundamental aim of our study is the comparison of metabolite activity based on the same amount of substance involved in radical scavenging calculated in micromoles rather than weight concentration. This approach provides an accurate way of comparing the radical scavenging activity, as it depends on the amount of substance and not on the weight concentration.

## 2. Results

### 2.1. Isolated Compounds from Lichens

Using the protocol below, the following pure compounds were isolated: gyrophoric acid (GA) from *Umbilicaria hirsuta*, evernic acid (EA) from *Evernia prunastri*, physodic acid (PSY), 3-hydroxyphysodic acid (3-OH), physodalic acid (PHYS), and atranorin (ATR) from *Hypogymnia physodes*.

### 2.2. NMR Spectra of Isolated Compounds

All studied compounds are known and were identified as atranorin, evernic acid, gyrophoric acid, physodic acid, 3-hydroxyphysodic acid, and physodalic acid by comparing their NMR (Table 1, Figure 1, Figure 2 and Figure 3 and Figures S1–S24) and IR spectroscopic data with those reported in the literature [16]. Because the available NMR data for metabolites were a ^1^H NMR spectrum less than 600 MHz, we engaged in the full assignment of ^1^H and ^13^C resonances for all compounds.

### 2.3. Antioxidant Screening Activity

**Figure 4:**
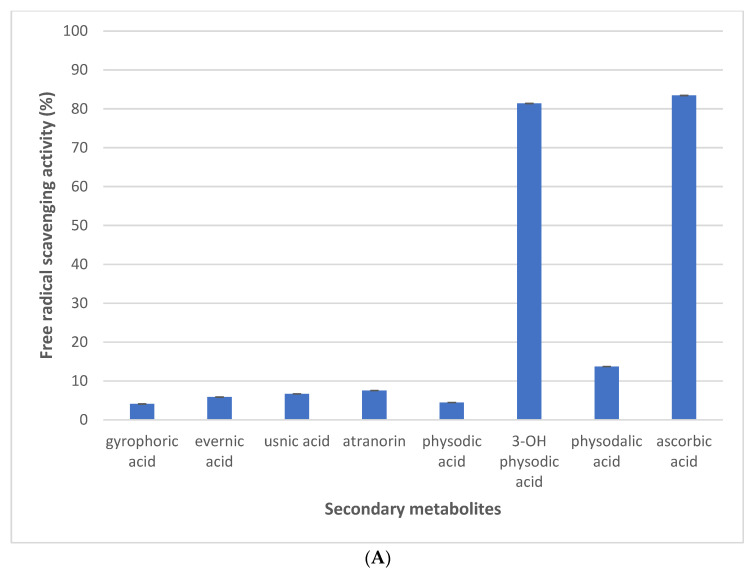

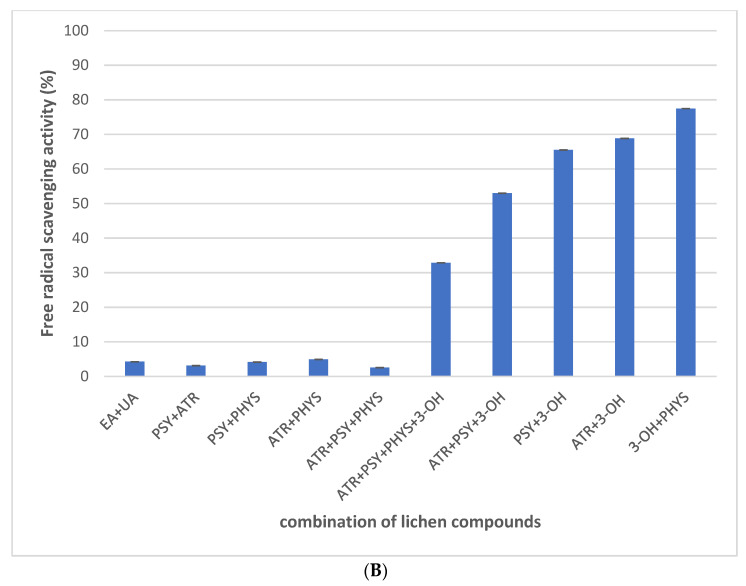
(**A**): Free radical scavenging activity of lichen metabolites and ascorbic acid as reference compound (*n* = 3); (**B**): free radical scavenging activity showed by a combination of lichen compounds (*n* = 3). Abbreviations mean: EA (evernic acid), UA (usnic acid), PSY (physodic acid), ATR (atranorin), PHYS (physodalic acid), 3-OH (3-hydroxyphysodic acid.

The free radical scavenging activity of the lichen compounds, as well as their combinations, are shown in Figure 4A,B. Single lichen compounds, such as gyrophoric acid, evernic acid, usnic acid, atranorin, physodic acid, and physodalic acid, showed scavenging radicals between 4 and 13%. The stronger potential of all isolated compounds showed 3-hydroxyphysodic acid, where the scavenging of free radicals was around 81%, while one of the most known antioxidants, ascorbic acid, showed scavenging of free radicals at 83% (Figure 4A).

The combination of secondary metabolites showed a similar trend in the presence of 3-hydroxyphysodic acid. Lichen compounds whose combination didn′t contain 3-hydroxyphysodic acid showed free radical scavenging activity between 2 and 4%. Combinations in which 3-hydroxyphysodic acid was present showed an increasing trend in the scavenging of free radicals from 32 to 77% (Figure 4B).

**Figure 5:**
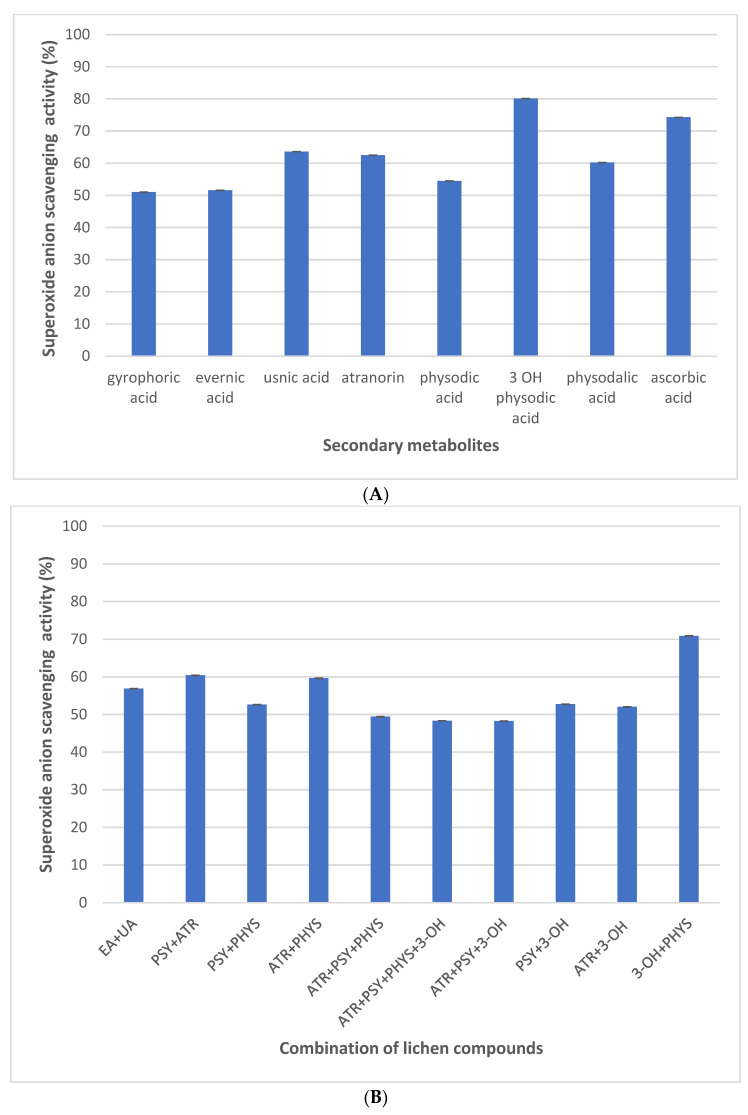
(**A**): Superoxide anion scavenging activity of lichen metabolites (*n* = 3) and ascorbic acid was used as a reference compound; (**B**): superoxide anion scavenging activity showed via combination of lichen compounds (*n* = 3). Abbreviations mean: EA (evernic acid), UA (usnic acid), PSY (physodic acid), ATR (atranorin), PHYS (physodalic acid), 3-OH (3-hydroxyphysodic acid.

The superoxide anion radical scavenging activity of the lichen compounds, as well as their combinations, are shown in Figure 5A,B. Single lichen compounds, such as gyrophoric acid, evernic acid, usnic acid, atranorin, physodic acid, and physodalic acid, showed superoxide anion scavenging radicals between 51 and 63%, which was around 10 times higher than in a previous free radical scavenging test. The strongest potential of all the isolated compounds showed again in those containing 3-hydroxyphysodic acid, where the scavenging of superoxide anion radicals was around 80%, while ascorbic acid showed scavenging activity at around 74% (Figure 5A).

The combination of secondary metabolites showed an average of superoxide anion scavenging, where the 3-hydroxyphysodic acid again showed the strongest potential. The combination of secondary compounds showed an average between 48 and 70%. Here, the scavenging of 3-hydroxyphysodic acid depends on concentration, which was added to the mixture with other secondary metabolites (Figure 5B).

## 3. Discussion

Water, methanol, and acetone extracts of many lichens from different environments have been tested for antioxidant activity. From these studies, a significant amount yielded positive results, exhibiting notable antioxidant activity, which means they performed better than, as well as, or slightly worse than BHT, BHA, or ascorbic acid as the positive control. Lichens from which the extract was made were, for example, *Lasallia pustulata*, *Hypogymnia physodes*, *Parmelia sulcata*, *Flavoparmelia caperata*, *Cladonia furcata*, *Peltigera rufescens*, *Cetraria islandica*, *Usnea ghattensis*, *Punctelia subrudecta*, and more [17,18,19,20,21]. Lichen extracts contain numerous groups of substances, such as carotenoids, carbohydrates, and products of acetyl-malonate pathways, such as depsides, depsidones, dibenzofurans, chromones, xanthones, and anthraquinones [22]. All mentioned substances have some antioxidant properties, and, in the studies above, a correlation of antioxidant activities with total phenolic content was not always found [18]. Antioxidant activity of phenolic compounds depends not only on the number of hydroxyl groups, but on many other factors, including the reactivity of hydroxyl groups, solvent (H-bonding), stability of aryloxy radical, steric factors, etc. [23].

This means that extracts with high antioxidant activity are not necessarily a reliable indicator of the antioxidant activities of particular secondary metabolites, and vice versa, as there might not be a sufficient concentration of the antioxidant active substance.

There are not so many reports with pure lichen substances because the isolation is very time-consuming and difficult. During the isolation of secondary metabolites from the chosen lichens by column chromatography, we encountered several issues, which brought us to design our practical procedures. Concerning solvents for mobile phases, we tried to use diethyl ether as the non-polar solvent, but, because of its high volatility that eventually changes the mobile phase ratio, as well as health issues, we chose cyclohexane instead. We also deduced that ethyl acetate and acetic acid are reliable mobile phase constituents for our purposes based on their physical properties (boiling point, polarity, volatility, etc.). Acetic acid helps to avoid the peak tailing in chromatography. In some cases with a similar polarity of substances (3-hydroxyphysodic acid and physodic acid), subsequent separation is required. Another observation during the separation by column chromatography was that undissolved compounds from the extract in our mobile phase led to an overlapping of metabolites. Because the raw extract contains many pigments (chlorophylls, carotenoids), the rinsing by cyclohexane seems to be helpful for reducing their presence. The polar character of compounds is very similar, which makes separation more complicated. Mostly the major compounds are possible to isolate in such amounts, which can be used for further studies. Bioactive substances isolated from lichens, such as atranorin, gyrophoric acid [24], usnic acid [20,25], and physodic acid, were tested due to their antioxidant activity. The antioxidant potential of physodalic acid was proven, but only as an compound in the tested extract from lichen *E. prunastri* [26].

Given the previous fact, we chose to examine the metabolites of atranorin, physodic acid, physodalic acid, 3-hydroxyphysodic acid, evernic acid, usnic acid, and gyrophoric acid calculated by amount of substance. All named secondary metabolites were already tested with the exception of 3-hydroxyphysodic acid [24,27,28].

This was because of the different molar weights of our studied metabolites, as well as of the ascorbic acid. We also provided screening of certain metabolite’s combinations of free radical scavenging and superoxide anion scavenging, which have not been examined yet.

Concerning the results of DPPH scavenging activity, we found out that at our chosen concentration, only 3-hydroxyphysodic acid and combinations containing this metabolite had a significant scavenging effect, and, when tested alone, results were even comparable to ascorbic acid (Figure 4). In the second antioxidant assay, the scavenging of superoxide anion radicals, all of our metabolites exhibited significant antioxidant activity ranging from 50 to 60% inhibition, again with 3-hydroxyphysodic acid with the highest activity at 80%, surpassing that of ascorbic acid (Figure 5).

**Figure 6:**
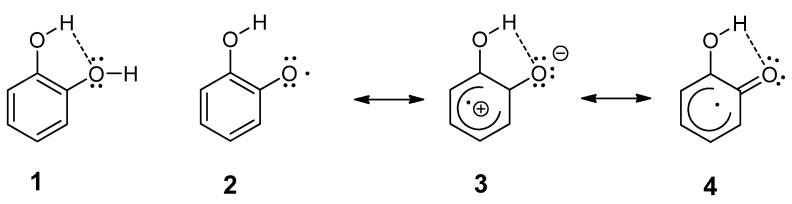
It is postulated that the increased stabilization of the semiquinone radical **2**, compared to that of the parent **1**, is due to the increase in strength of the intramolecular H-bond induced by dipolar **3**’s and keto-enol **4**’s contributions to the radical’s structure [29].

In our experiment 3-hydroxyphysodic acid showed the best free radical scavenging and superoxide radical scavenging activities. The 3-hydroxyphysodic acid contains three hydroxyl groups, similar to gyrophoric acid and usnic acid, but it contains a catechol fragment as well. It is known that intramolecular hydrogen bonding in the catechol ring system has a pronounced effect on antioxidant activity (Figure 6) by stabilisation of the *o*-semiquinone radical [29]. In these compounds, the hydroxyl group supports the homolytic cleavage of the neighboring O–H bond and enables the formation of a hydrogen bond with a formed phenoxy radical [30].

Our findings correspond with work on the kinetics and antioxidant mechanisms of depsidones of *Ramalina* sp. They concluded that depsidones are not good RO$O^{•}$ scavengers in either polar or nonpolar environments, but they are excellent H$O^{•}$ and $O_{2}^{•\;-}$ scavengers in aqueous media [31]. From our examined metabolites, physodic, 3-hydroxyphysodic, and physodalic acid are depsidones; however, atranorin and evernic acid are didepsides, gyrophoric acid is tridepside, and usnic acid belongs to dibenzofurans. Considering these metabolites exhibited very similar activities across the antioxidant assays, we might speculate that they are reacting via the same mechanism as the depsidones, despite their differences in molecular structure. With regard to the importance of an aqueous environment for optimal antioxidant activity, as they are mostly ionized [31], we dissolved our metabolites in 5% DMSO solution, instead of methanol because it has weak, but still acidic, hydrogen that might cause some shift in the chemical balance, thereby changing the ratios of both ionized and non-ionized forms.

Our supplementary investigation will be a screening of other metabolites in this manner, since we consider it to be the most accurate way to compare antioxidant activities, as was mentioned above. The ratios for combinations of our metabolites were set to roughly mimic the actual content of the metabolites in lichen extracts. The precise measurements of the secondary metabolites and their concentration in lichens can be completed by HPLC. Their amount varies and depends on the environment as well as the chemotypes. This approach might elucidate how much of extract activity is dependent on the content of secondary metabolites.

## 4. Material and Methods

### 4.1. Collection of Lichens

Lichen *Hypogymnia physodes* was collected from barks of *Picea abies* at Kojšová hoľa in Volovské vrchy (Košice, Slovakia) during September 2019. *H. physodes* was determined by Dr. Goga to be a lichen specimen with the number KO36.716. Lichen *Evernia prunastri* was collected from branches of *Prunus spinosa* at Zemplínske vrchy, Vlčia hora (Cejkov, Slovakia) during December 2019. *E. prunastri* was determined by Dr. Dudáš to be a lichen specimen with the number KO32358. Lichen *Umbilicaria hirsuta* was collected from andesite rock at Sninský kameň, Vihorlat (Sninský kameň, Slovakia) during August 2019. The lichen specimen was determined by Dr. Goga with the number KO35121. All specimens are deposited in herbarium of P.J. Šafárik in Košice, Slovakia.

#### Isolation of Lichen Compounds by Thin-Layer Chromatography (TLC) and Column Chromatography

For isolation of the compounds from lichen *E. prunastri* and *H. physodes*, the following conditions were used. Lichen thalli (10 g, dry weight) were put into a glass beaker and rinsed with 500 mL of acetone for the extraction of secondary metabolites, according to Solhaug and Gauslaa [32]. The lichen thalli were mixed with a magnetic stirrer for 24 h. The acetone was evaporated by a rotary evaporator, and the powder of the secondary metabolites was stored for further experiments in the fridge at 4 °C. For isolation of the secondary metabolites from *E. prunastri* extract, the mobile phase of cyclohexane/ethyl acetate/acetic acid (in ratio 5:1:0.4) was used. For isolation of lichen compounds from *H. physodes* extract, the mobile phase of cyclohexane/ethyl acetate/acetic acid (in ratio 2:1:0.2) was used. The isolation of gyrophoric acid from lichen *U. hirsuta* was performed according to Goga et al. [33].

For TLC analysis of the lichen extracts, the aluminium plates Merck silica gel 60 F_254_ were used. Visualisation of the TLC spots was performed using UV light (λ = 254 nm) and an ethanolic solution of vanillin with further heating.

Column chromatography was performed using the silica gel Kieselgel 60 (0.040–0.063 mm, 230–400 mesh) as a stationary phase, and organic solvents in the p.a. purity were used as a mobile phase. For isolation and purification of certain secondary metabolites, a preparative chromatography via a glass column (2 cm diameter) filled with silica gel (30 cm height) was used and washed with the appropriate mobile phase. Fractions (4 mL) were collected and identified using TLC. Fractions containing the desired products were collected and evaporated under reduced pressure on a rotary evaporator to yield solids. The structure of isolated products was confirmed using one- and two-dimensional NMR techniques.

### 4.2. Identification of Lichen Substances by NMR

NMR spectra were measured using the Varian VNMRS 600 MHz spectrometer equipped with an OneNMR 5 mm probe. NMR spectra were calibrated using solvent residual signals as references. The compounds were dissolved in 500 μL of DMSO-d_6_ (Merck) or CD_3_OD-d_4_ (Merck). The ^1^H and ^13^C NMR spectra (acquired at 599.86 MHz and 150.85 MHz, respectively) were measured at a temperature of 298.15 K. Chemical shifts (*δ*) are expressed in parts per million (ppm), and coupling constants (*J*) are reported in Hertz (Hz). NMR experiments were performed using standard Varian pulse sequences.

### 4.3. Free Radical Scavenging Activity by DPPH

The free radical scavenging activity of isolated compounds, as well as their mixtures, were measured using 1,1-diphenyl-2-picryl-hydrazil (DPPH). Usnic acid (UA, Aldrich Company 329967, purity 98%) was used as a synthetic compound, and, as a reference compound, ascorbic acid (AA, Aldrich Company A92902, purity 99%) was used. The antioxidant activity via this method is described in several studies, and here, we slightly modify it [17,34,35]. The secondary metabolites were weighed based on the amount of substance, dissolved in 5% DMSO, and served as a stock solution for further screening of the antioxidant activity. For DPPH, 0.2 µmol/1 mL of pure substances was used. For mixtures of compounds the following ratios were used: EA/UA 1:1; PHY/ATR 1:1; 3-OH/PHYS 1:1; PHY/3-OH 3:1; ATR/3-OH 3:1; ATR/PHY/3-OH 2:2:1; PHY/PHYS 3:1; ATR/PHYS 3:1; ATR/PHY/PHYS 2:2:1; and ATR/PHY/3-OH/PHYS 4:4:1:1. Secondary metabolites were sonicated and vortexed until the compounds were fully dissolved. DPPH (0.1 mM) was dissolved in MeOH. Falcone tubes (15 mL) were filled with 1 mL of dissolved secondary metabolites, as well as the ratios of their mixtures, and then with 2 mL of DPPH to reach a final volume of 3 mL. The samples were incubated at laboratory temperature in the dark for 30 min. After the incubation, the absorbance of the samples was measured at 517 nm in a spectrophotometer (multi-detection microplate reader; the Synergy HT, BioTek). A solution of 5% DMSO (1 mL) in MeOH (2 mL) was used as a blank control. The DPPH radical concentration was calculated by the Equation (1):

Free radical scavenging activity (%) = (A_0_ − A_1_/A_0_) ∗ 100, (1)

where A_0_ was the absorbance of negative control, and A_1_ was the absorbance of the reaction mixture of our samples.

### 4.4. Superoxide Anion Scavenging Activity

The superoxide anion scavenging activity of our isolated compounds, as well as their mixtures, was measured according to the Nishimiki method [36]. The method was also used in another study [7] of antioxidant activity, and here, we slightly modified it. The secondary metabolites were weighed based on the amount of substance, dissolved in 5% DMSO, and served as a stock solution. For superoxide anion scavenging activity, 0.963 µmol/0.1 mL of pure substances was used. Mixtures of the same rations as for the DPPH method were used. Secondary metabolites were sonicated and vortexed until the compounds were fully dissolved. A portion (100 µL) of each prepared sample was mixed with 1 mL of NADH (468 µM of nicotinamide adenine dinucleotide solution in 0.1 M phosphate buffer with pH 7.4) and 1 mL of NBT (156 µM of nitroblue tetrazolium solution in 0.1 M phosphate buffer with pH 7.4). The reaction started by adding of 100 µL of PMS (60 µM of phenazine methosulfate solution dissolved in 0.1 M phosphate buffer with pH 7.4). The mixture was incubated at laboratory temperature in the dark for 5 min. After that, the absorbance was measured at 560 nm in a spectrophotometer (multi-detection microplate reader; the Synergy HT, BioTek). The superoxide anion concentration was expressed as a percentage by the Equation (2):

Superoxide anion scavenging activity (%) = (A_0_ − A_1_/A_0_) ∗ 100, (2)

where A_0_ was the absorbance of negative control, and A_1_ was the absorbance of reaction mixture of our samples.

## 5. Conclusions

In our study, we show how to isolate various secondary metabolites from lichens and confirmed their structures by one- and two-dimensional NMR methods. These metabolites can serve for other biological activities, instead of antioxidant activity, which was tested in our manuscript. We show novelty in the calculation of antioxidant activity, which rests in recalculation of the tested compounds based on amount of substance. We assume that this recalculation is more precise because the stoichiometry of compounds was considered. In our experiment, we tested the antioxidant properties of isolated compounds, as well as their mixtures, via two different methods. The scavenging of free radicals showed different activity when compared with superoxide anion radical scavenging activity. The most scavenging activity was demonstrated by the lichen secondary metabolite 3-hydroxyphysodic acid, which was tested as a pure substance for the first time here. Consequently, the tested mixtures of metabolites containing the 3-hydroxyphysodic acid showed stronger antioxidant potential. Kinetic studies of the lichen secondary metabolite’s reactions need further investigations and are planned.

## Figures and Tables

**Figure 1:**
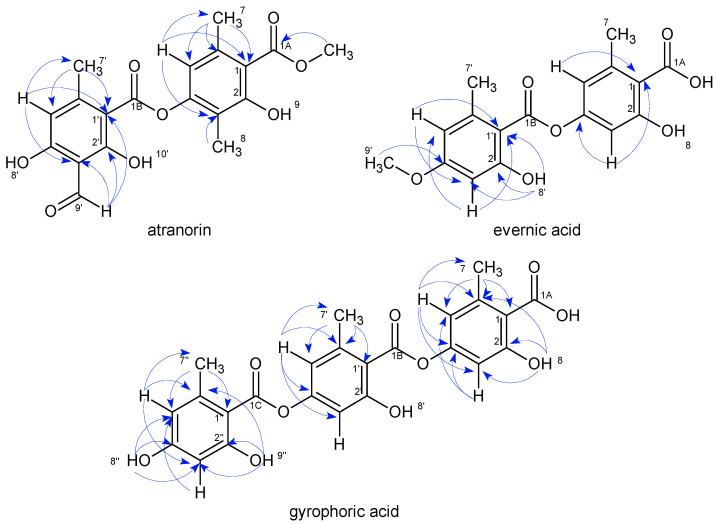
Chemical structures of depsides, atranorin, evernic acid, and gyrophoric acid along with their key HMBC correlations.

**Figure 2:**
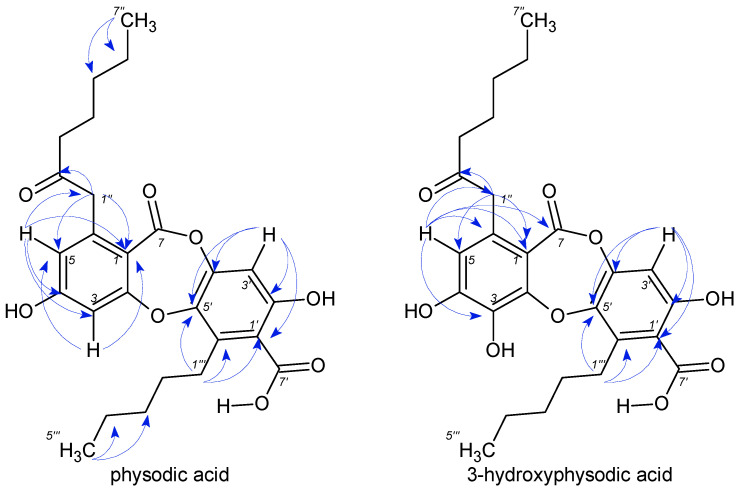
Chemical structures of depsides, physodic, and 3-hydroxyphysodic acid along with their key HMBC correlations.

**Figure 3:**
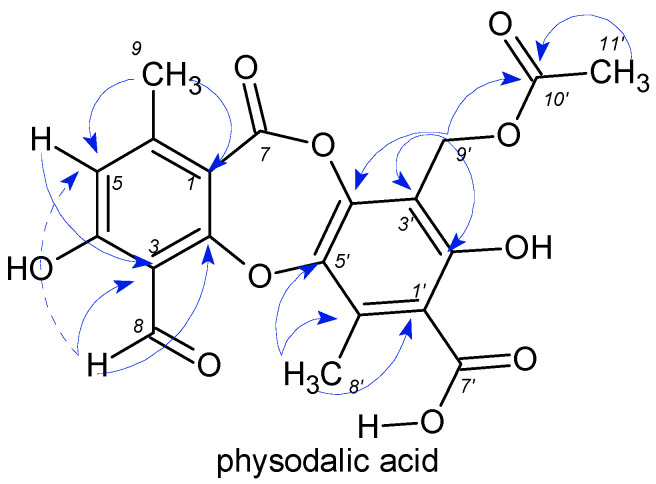
Chemical structure of depside physodalic acid along with its key HMBC correlations.

**Table 1:** ^1^H (600 MHz) and ^13^C (150 MHz) NMR data of atranorin, evernic, gyrophoric acid, physodic acid, 3-hydroxyphysodic acid, and physodalic acid in DMSO-d_6_.

	Atranorin		Evernic Acid		Gyrophoric Acid		Physodic Acid		3-Hydroxyphysodic Acid		Physodalic Acid	
	*δ*_H_ (mult, *J*)	*δ*_C_	*δ*_H_ (mult, *J*)	*δ*_C_	*δ*_H_ (mult, *J*)	*δ*_C_	*δ*_H_ (mult, *J*)	*δ*_C_	*δ*_H_ (mult, *J*)	*δ*_C_	*δ*_H_ (mult, *J*)	*δ*_C_
**1A**		169.7		170.6		170.4						
**1**		115.3		116.8		138.0		113.5		112.1		108.6
**2**		157.4		159.3		156.3		164.5		150.0		166.5
**3**		116.3	6.59 (d, 2.3)	107.3	6.68 (d, 2.1)	107.2	6.64 (d, 2.4)	106.9		134.2		112.6
**4**		151.4		152.1		152.1		163.9		150.3		167.9
**5**	6.65 (s)	115.7	6.56 (d, 2.3)	114.4	6.67 (d, 2.0)	114.2	6.55 (d, 2.3)	118.2	6.53 (s)	114.7	6.55 (s)	119.3
**6**		136.5		139.7		118.0		143.1		129.3		150.3
**7**	2.35 (s)	21.1	2.38 (s)	21.2	2.37 (s)	19.3		164.7		163.4		162.1
**8**	2.04 (s)	9.3			10.47 (s)						10.52 (s)	190.8
**9**	10.52 (s)										2.35 (s)	21.4
**10**	3.88 (s)	52.3										
**1B**		164.5		166.8		165.6						
**1** **′**		110.7		110.6		139.4		114.5		113.6		115.2
**2** **′**		161.5		159.3		158.5		159.5		156.9		162.8
**3** **′**		107.9	6.36 (d, 2.4)	99.0	6.64 (d, 2.2)	107.2	6.62 (s)	107.5	6.58 (s)	104.8		111.4
**4** **′**		163.4		162.1		152.1		149.1		146.7		145.0
**5** **′**	6.42 (s)	109.0	6.40 (d, 2.4)	108.1	6.61 (d, 2.1)	114.4		142.9		141.6		139.8
**6** **′**		149.0		139.7		117.3		138.9		137.7		133.6
**7** **′**	2.40 (s)	21.2	2.38 (s)	21.0	2.37 (s)	20.8		173.1		171.3		170.7
**8** **′**			10.40 (s)								2.64 (s)	14.2
**9** **′**	10.22 (s)	193.9	3.75 (s)	55.2							5.06 (s)	56.2
**10** **′**												170.5
**11** **′**											1.96 (s)	20.8
**1C**						167.1						
**1″**						140.1	3.97 (s)	48.8	3.84 (s)	46.3		
**2″**						159.9		209.6		208.3		
**3″**					6.24 (d, 2.3)	100.5	2.53 (t, 7.4)	43.2	2.50 (t, 7.4)	40.9		
**4″**						161.0	1.58 (m)	24.4	1.56 (m)	22.3		
**5″**					6.22 (d, 2.1)	109.8	1.29 (m)	32.5	1.28 (m)	30.3		
**6″**						108.5	1.34 (m)	23.5	1.32 (m)	21.4		
**7″**					2.35 (s)	21.2	0.91 (t, 7.2)	14.3	0.90 (t, 7.2)	12.1		
**8″**					10.00 (s)		3.15 (ddd, 10.8)	28.9				
**9″**					10.30 (s)		1.62 (m)	32.0				
**1‴**							1.49 (m)	33.4	3.41 (m)	26.2		
**2‴**							1.43 (m)	23.5	1.56 (m)	30.4		
**3‴**							0.96 (t, 7.4)	14.4	1.45 (m)	31.4		
**4‴**							3.15 (ddd, 10.8)	28.9	1.38 (m)	21.5		
**5‴**							1.62 (m)	32.0	0.92 (t, 7.2)	12.4		

## Data Availability

The data can be provided by the authors upon reasonable request.

## Supplementary Materials

The following supporting information can be downloaded at: https://www.mdpi.com/article/10.3390/plants11081077/s1, Figure S1. ^1^H NMR spectrum (600 MHz, DMSO-d_6_) of atranorin; Figure S2. ^13^C NMR spectrum (150 MHz, DMSO-d_6_) of atranorin; Figure S3. ^1^H,^13^C-HSQC NMR spectrum (600 MHz, 150 MHz, DMSO-d_6_) of atranorin; Figure S4. ^1^H,^13^C-HMBC NMR spectrum (600 MHz, 150 MHz, DMSO-d_6_) of atranorin; Figure S5. ^1^H NMR spectrum (600 MHz, DMSO-d_6_) of evernic acid; Figure S6. ^13^C NMR spectrum (150 MHz, DMSO-d_6_) of evernic acid; Figure S7. ^1^H,^13^C-HSQC NMR spectrum (600 MHz, 150 MHz, DMSO-d_6_) of evernic acid; Figure S8. ^1^H,^13^C-HMBC NMR spectrum (600 MHz, 150 MHz, DMSO-d_6_) of evernic acid; Figure S9. ^1^H NMR spectrum (600 MHz, DMSO-d_6_) of gyrophoric acid.; Figure S10. ^13^C NMR spectrum (150 MHz, DMSO-d_6_) of gyrophoric acid; Figure S11. ^1^H,^13^C-HSQC NMR spectrum (600 MHz, 150 MHz, DMSO-d_6_) of gyrophoric acid; Figure S12. ^1^H,^13^C-HMBC NMR spectrum (600 MHz, 150 MHz, DMSO-d_6_) of gyrophoric acid; Figure S13. ^1^H NMR spectrum (600 MHz, CD_3_OD-d_4_) of physodic acid; Figure S14. ^13^C NMR spectrum (150 MHz, CD_3_OD-d_4_) of physodic acid; Figure S15. ^1^H,^13^C-HSQC NMR spectrum (600 MHz, 150 MHz, CD_3_OD-d_4_) of physodic acid; Figure S16. ^1^H,^13^C-HMBC NMR spectrum (600 MHz, 150 MHz, CD_3_OD-d_4_) of physodic acid; Figure S17. ^1^H NMR spectrum (600 MHz, CD_3_OD-d_4_) of 3-hydroxyphysodic acid; Figure S18. ^13^C NMR spectrum (150 MHz, CD_3_OD-d_4_) of 3-hydroxyphysodic acid; Figure S19. ^1^H,^13^C-HSQC NMR spectrum (600 MHz, 150 MHz, CD_3_OD-d_4_) of 3-hydroxyphysodic acid; Figure S20. ^1^H,^13^C-HMBC NMR spectrum (600 MHz, 150 MHz, CD_3_OD-d_4_) of 3-hydroxyphysodic acid; Figure S21. ^1^H NMR spectrum (600 MHz, DMSO-d_6_) of physodalic acid; Figure S22. ^13^C NMR spectrum (150 MHz, DMSO-d_6_) of physodalic acid; Figure S23. ^1^H,^13^C-HSQC NMR spectrum (600 MHz, 150 MHz, DMSO-d_6_) of physodalic acid; Figure S24. ^1^H,^13^C-HMBC NMR spectrum (600 MHz, 150 MHz, DMSO-d_6_) of physodalic acid.
