# Supplementary material for: A Comparative Study of Isolated Secondary Metabolites from Lichens and Their Antioxidative Properties
Source: Plants (Basel). 2022 Apr 15;11(8):1077. doi: 10.3390/plants11081077 (PMC9032407; doi:10.3390/plants11081077)
Supplement: Supplementary file 1 [file plants-11-01077-s001.zip › plants-1684041-supplementary.pdf]

plants

MDPI

Supplementary Information

A Comparative Study of Isolated Secondary Metabolites from Lichens and Their Antioxidative Properties

Ján Elečko[1], Mária Vilková[1], Richard Frenák[2], Deepi Routray[2], Dajana Ručová[2], Martin Bačkor[2,3], Michal Goga[2*]

[1] *NMR laboratory, Department of Chemistry, Faculty of Science, Pavol Jozef Šafárik University, Moyzesova 11, 040 01 Košice, Slovakia*

[2] *Department of Botany, Institute of Biology and Ecology, Faculty of Science, Pavol Jozef Šafárik University, Mánesova 23, 041 67 Košice, Slovakia*

[3] *Institute of Biotechnology, Faculty of Biotechnology and Food Sciences, Slovak University of Agriculture in Nitra, 94976, Nitra, Slovakia*

1. NMR spectra

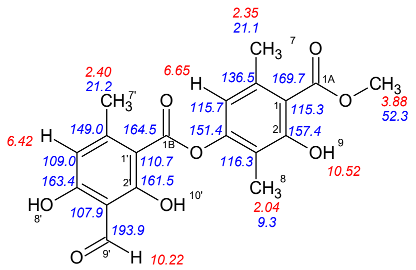

atranorin

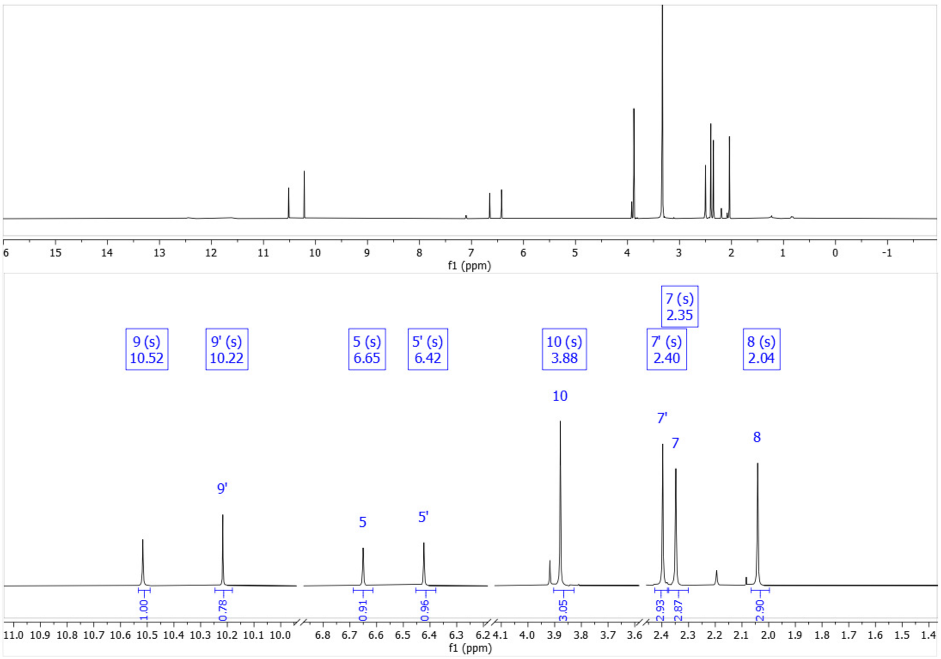

Figure S1. ^{1}H NMR spectrum (600 MHz, DMSO-d_6) of atranorin.

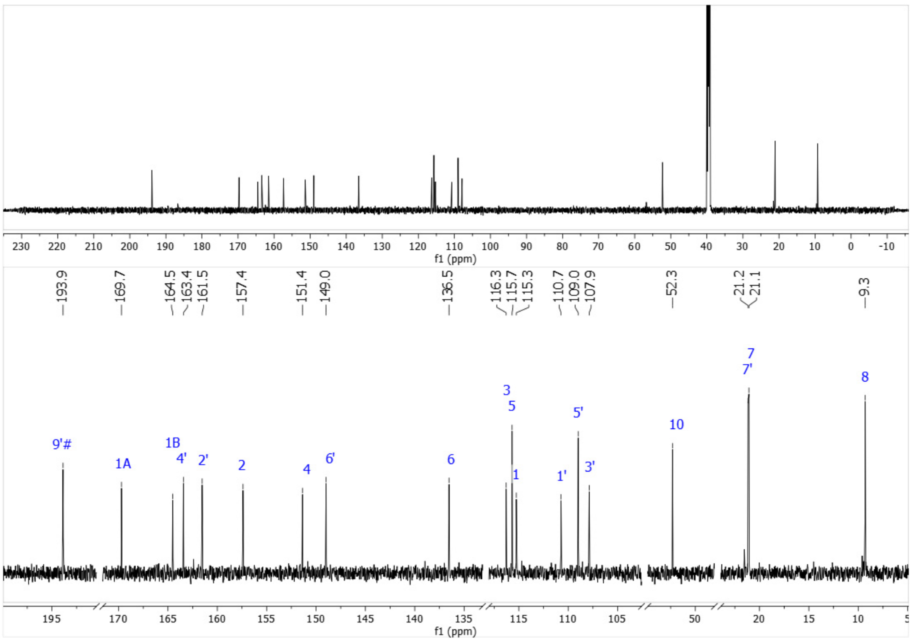

Figure S2. ^{13}C NMR spectrum (150 MHz, DMSO-d_6) of atranorin.

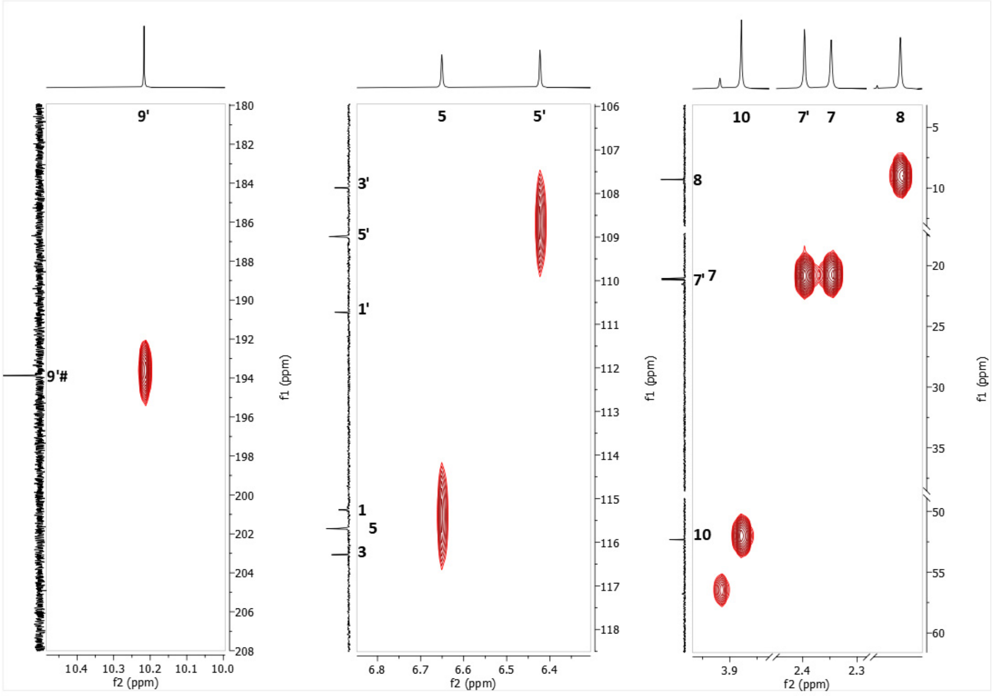

Figure S3. ^{1}H,^{13}C-HSQC NMR spectrum (600 MHz, 150 MHz, DMSO-d$_6$) of atranorin.

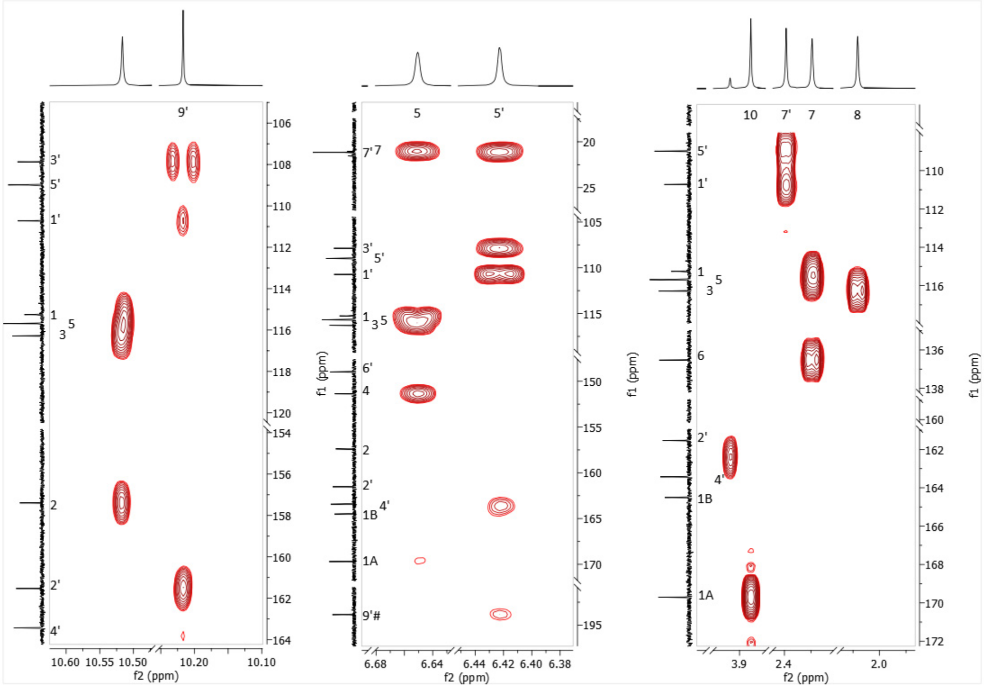

Figure S4. ^{1}H,^{13}C-HMBC NMR spectrum (600 MHz, 150 MHz, DMSO-d$_6$) of atranorin.

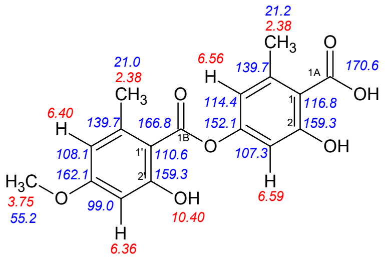

evernic acid

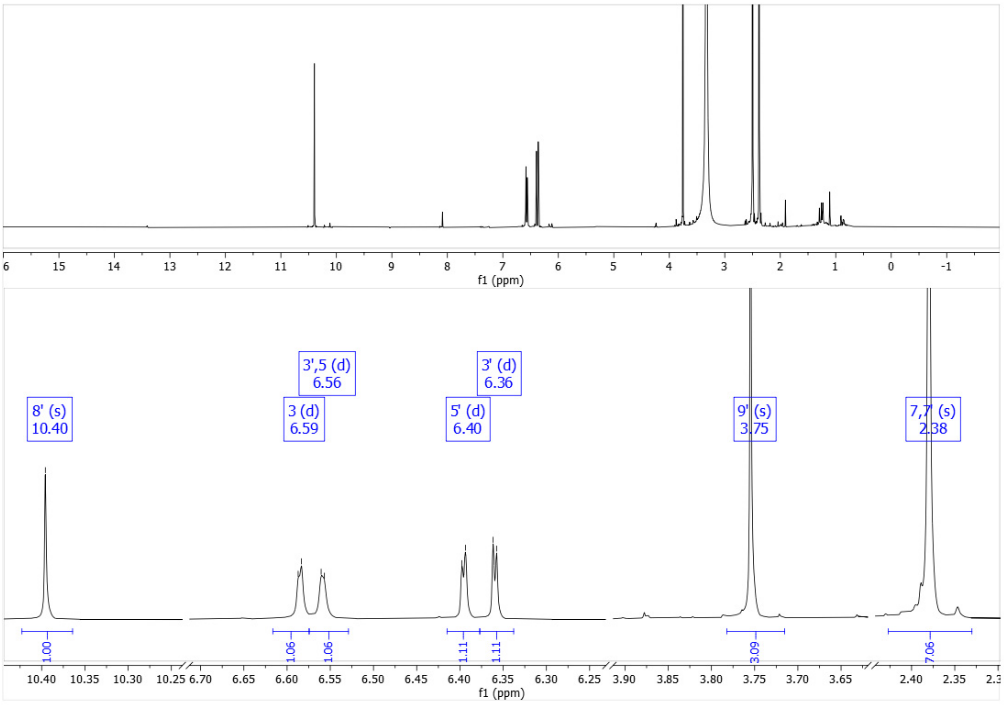

Figure S5. ^{1}H NMR spectrum (600 MHz, DMSO-d_6) of evernic acid.

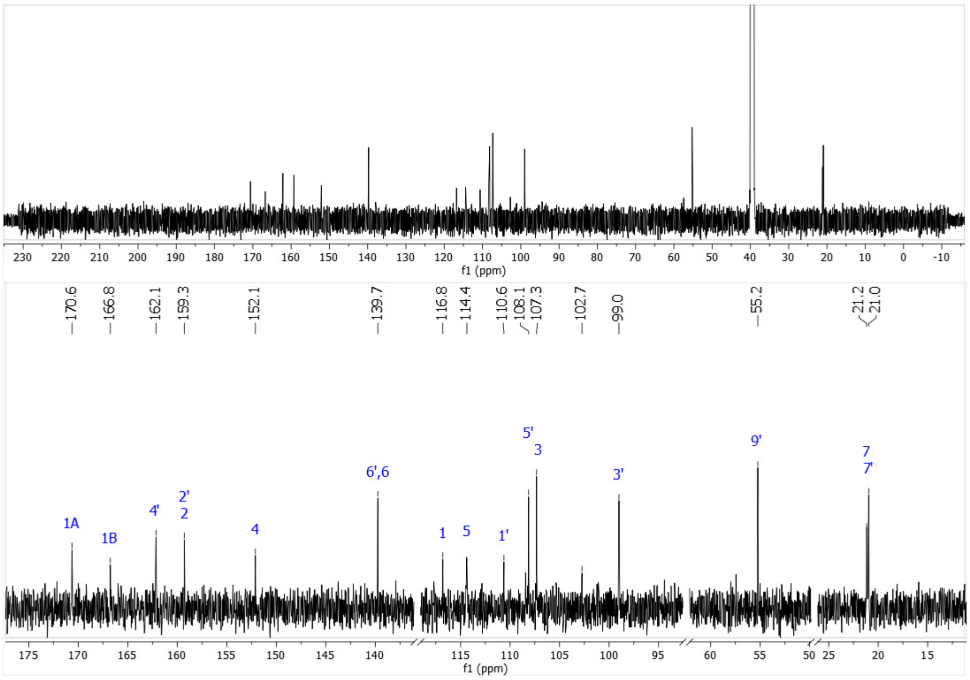

Figure S6. ^{13}C NMR spectrum (150 MHz, DMSO-d_6) of evernic acid.

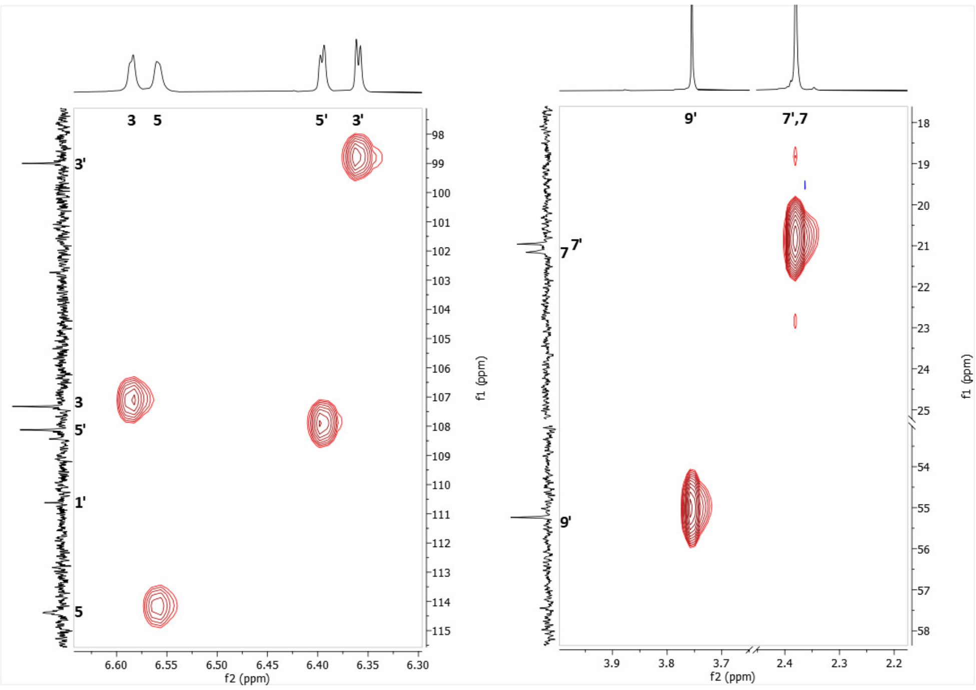

Figure S7. ^{1}H,^{13}C-HSQC NMR spectrum (600 MHz, 150 MHz, DMSO-d_6) of evernic acid.

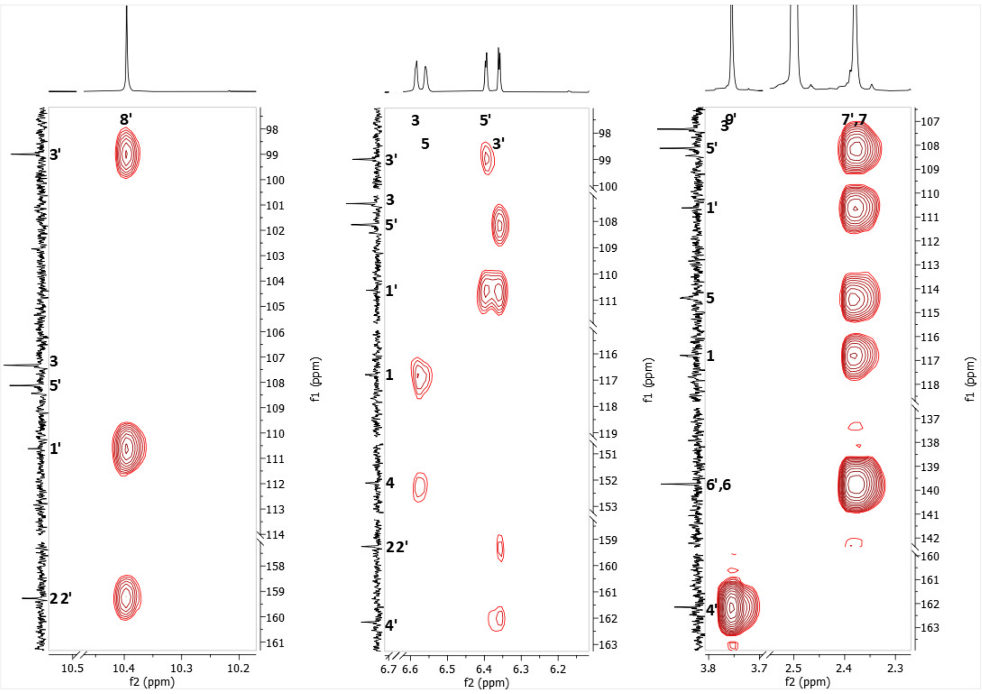

Figure S8. ^{1}H,^{13}C-HMBC NMR spectrum (600 MHz, 150 MHz, DMSO-d_6) of evernic acid.

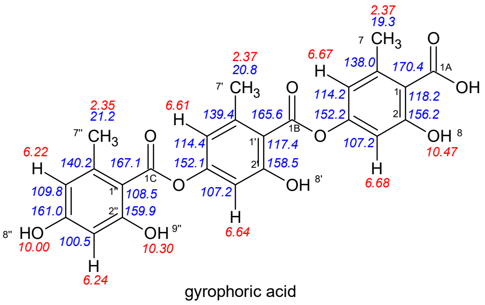

gyrophoric acid

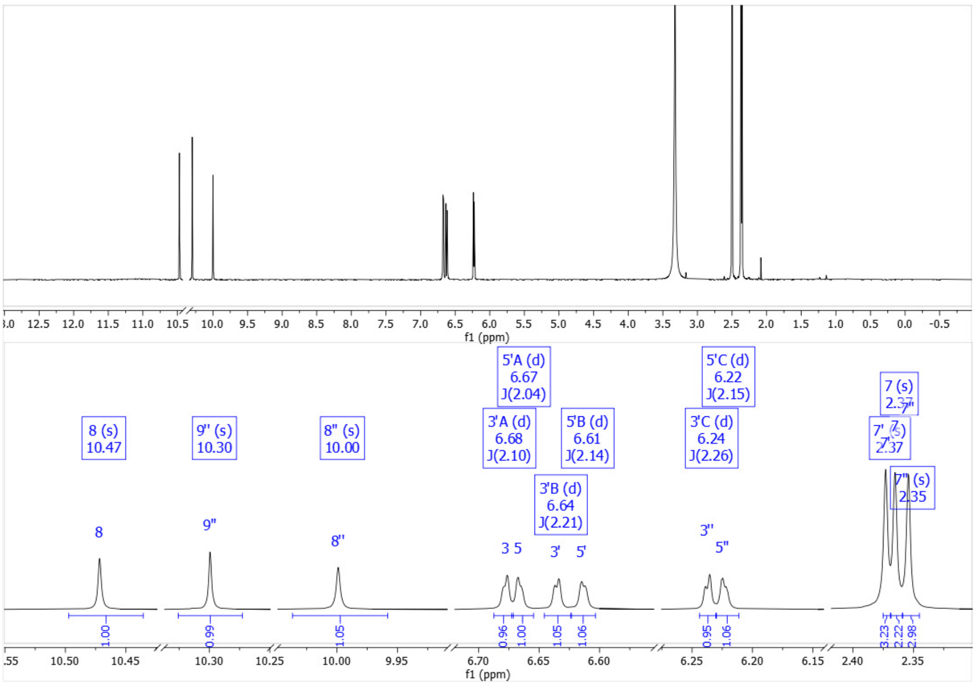

Figure S9. ^{1}H NMR spectrum (600 MHz, DMSO-d$_6$) of gyrophoric acid.

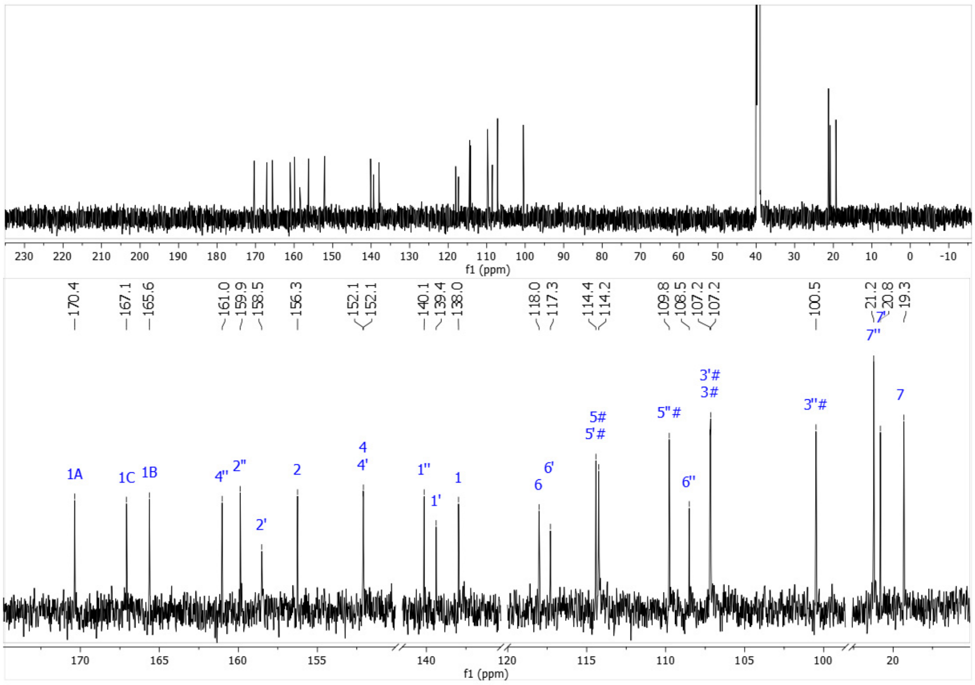

Figure S10. ^{13}C NMR spectrum (150 MHz, DMSO-d$_6$) of gyrophoric acid.

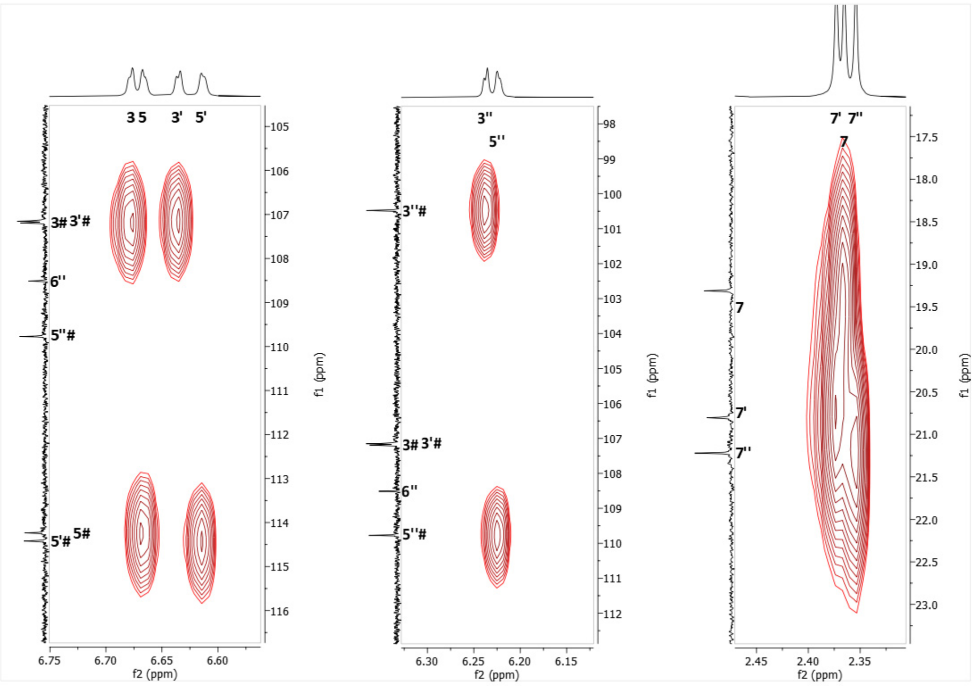

Figure S11. ^{1}H,^{13}C-HSQC NMR spectrum (600 MHz, 150 MHz, DMSO-d_6) of gyrophoric acid.

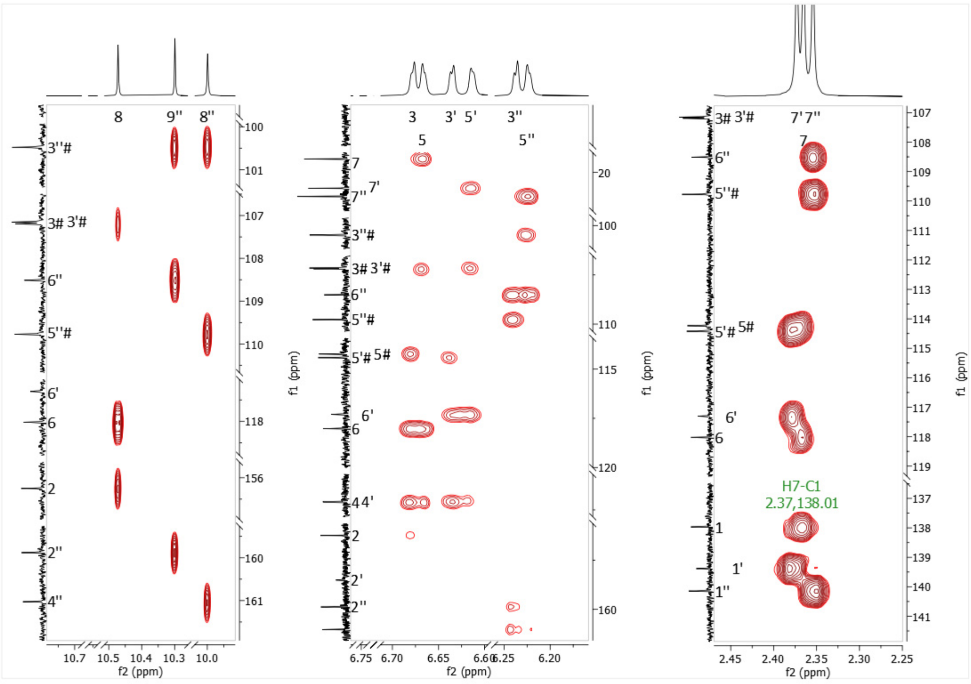

Figure S12. ^{1}H,^{13}C-HMBC NMR spectrum (600 MHz, 150 MHz, DMSO-d_6) of gyrophoric acid.

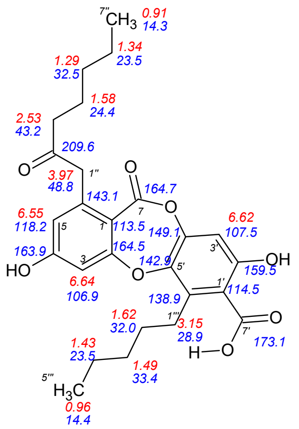

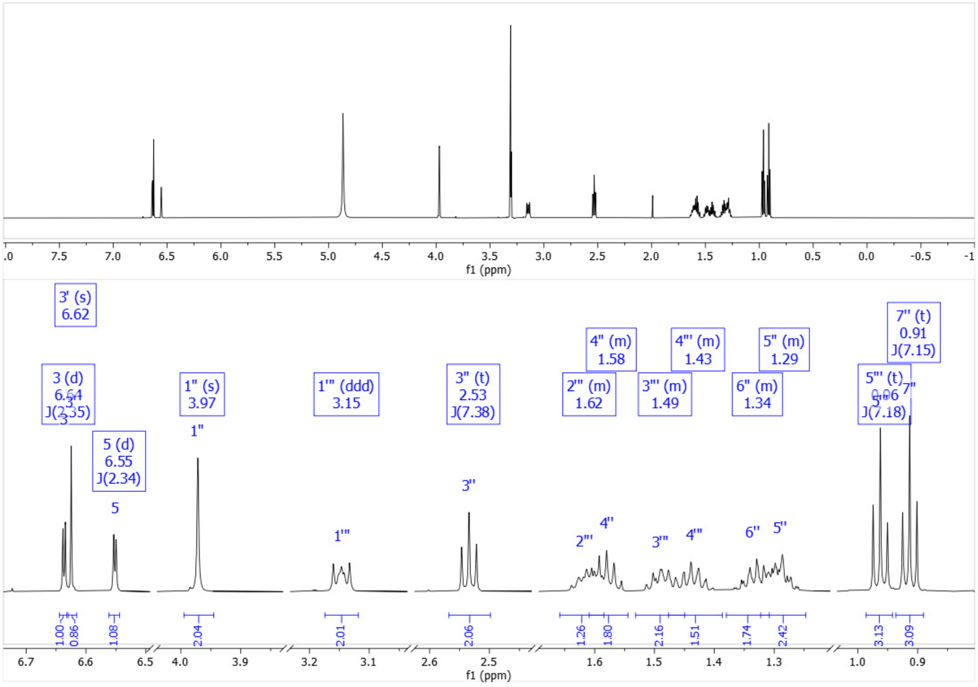

Figure S13. ^{1}H NMR spectrum (600 MHz, CD_3OD-d_4) of physodic acid.

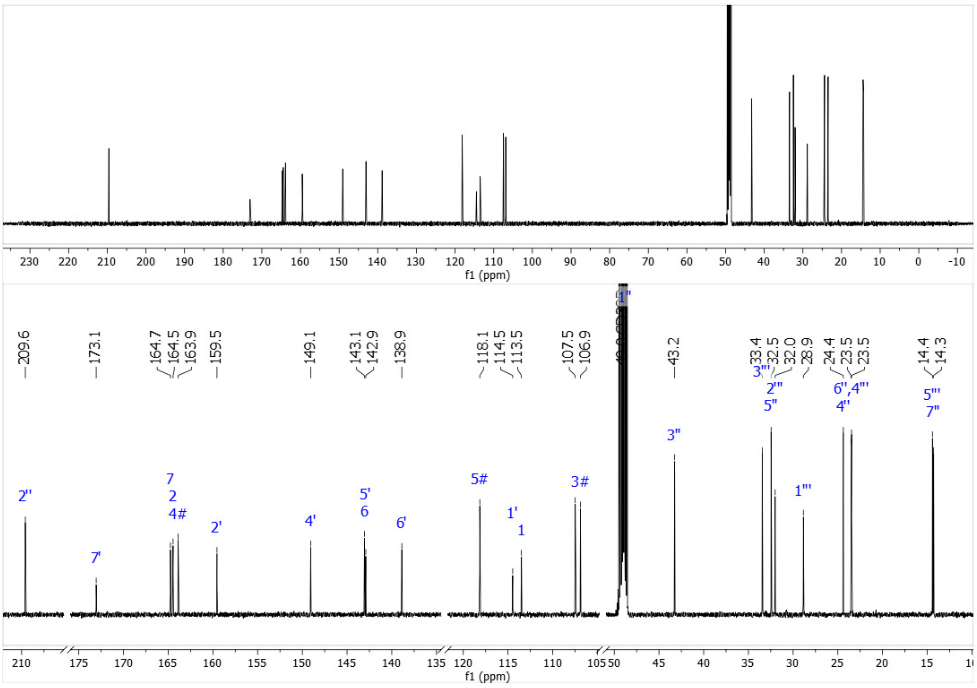

Figure S14. ^{13}C NMR spectrum (150 MHz, CD_3OD-d_4) of physodic acid.

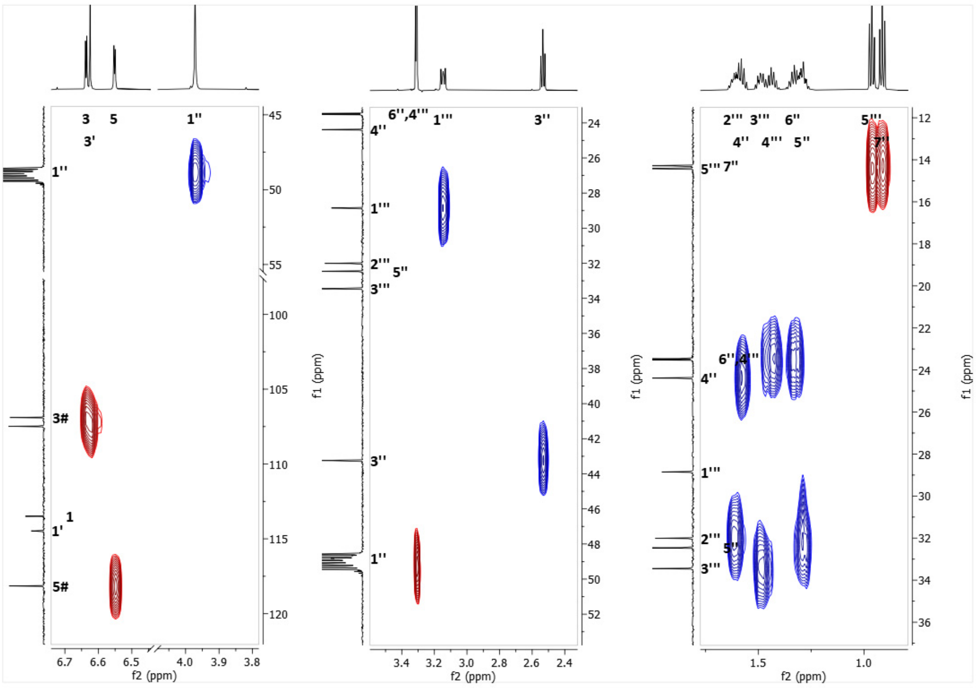

Figure S15. ^{1}H,^{13}C-HSQC NMR spectrum (600 MHz, 150 MHz, CD_3OD-d_4) of physodic acid.

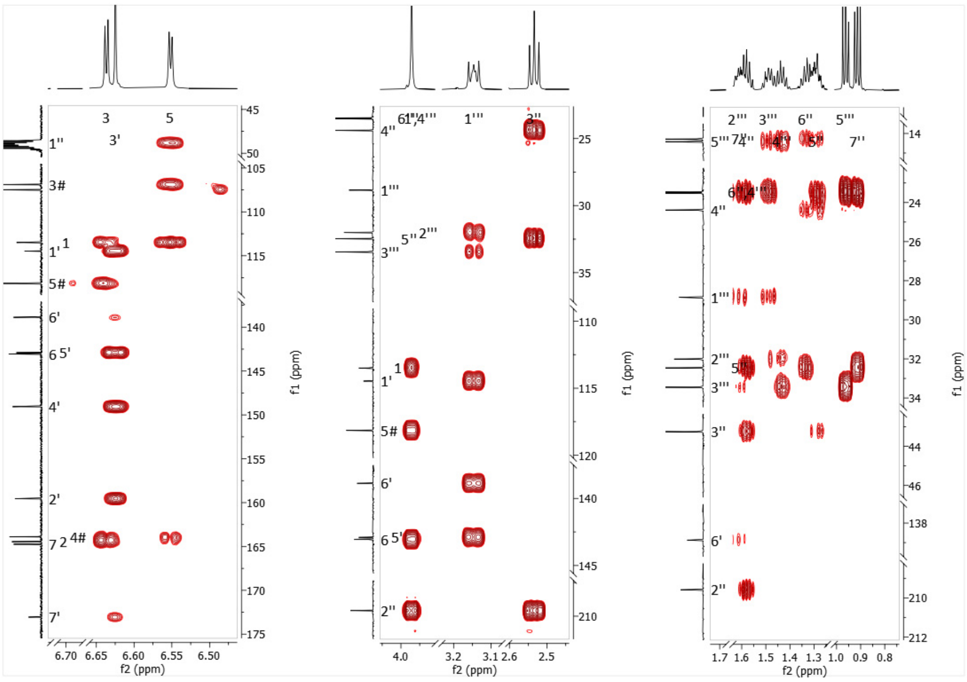

Figure S16. ^{1}H,^{13}C-HMBC NMR spectrum (600 MHz, 150 MHz, CD_3OD-d_4) of physodic acid.

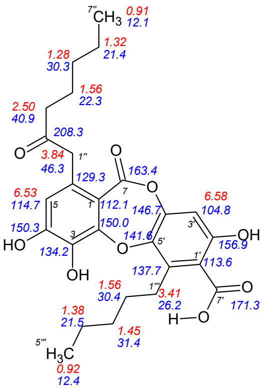

3-hydroxyphysodic acid

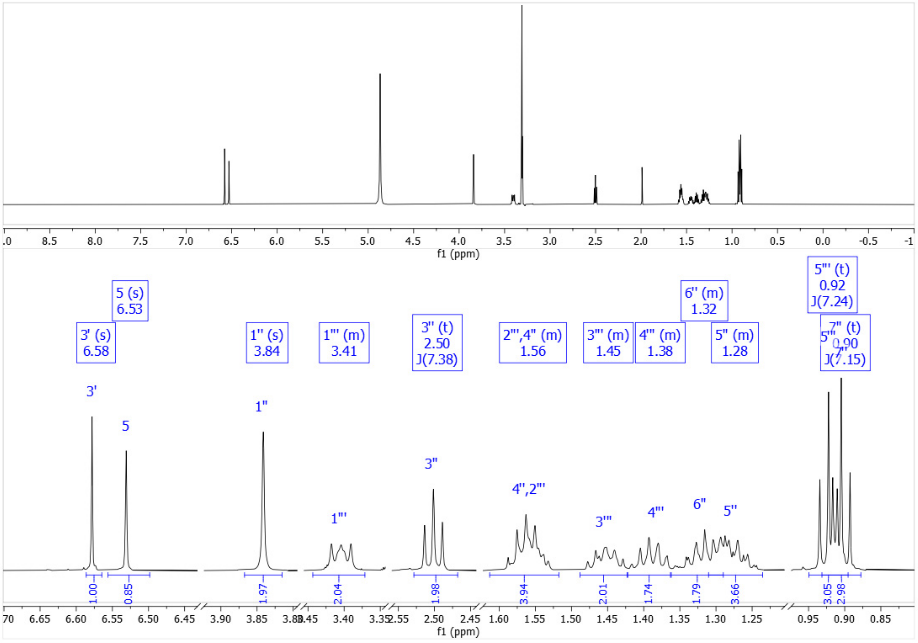

Figure S17. ^1H NMR spectrum (600 MHz, CD_3OD-d_4) of 3-hydroxyphysodic acid.

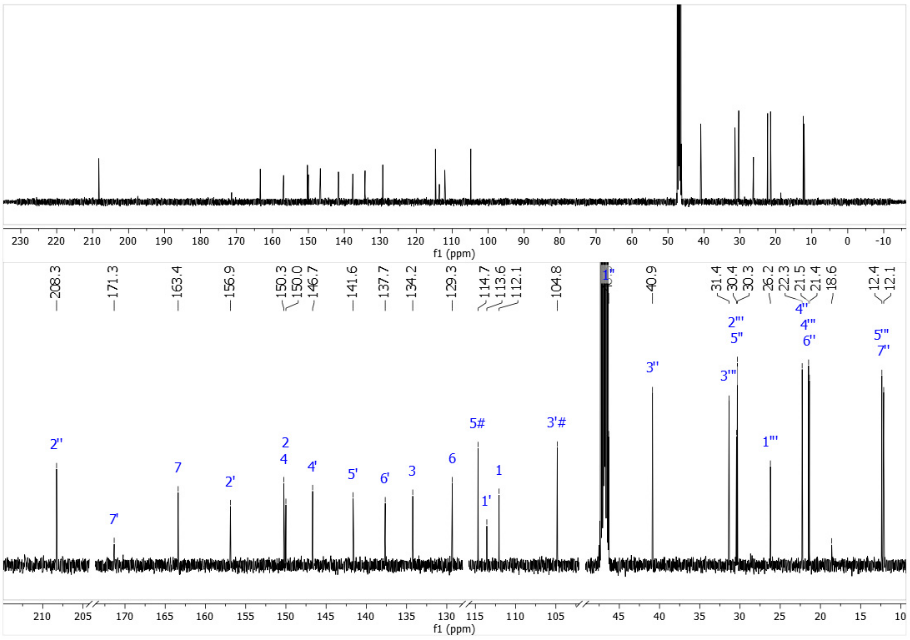

Figure S18. ^{13}C NMR spectrum (150 MHz, CD_3OD-d_4) of 3-hydroxyphysodic acid.

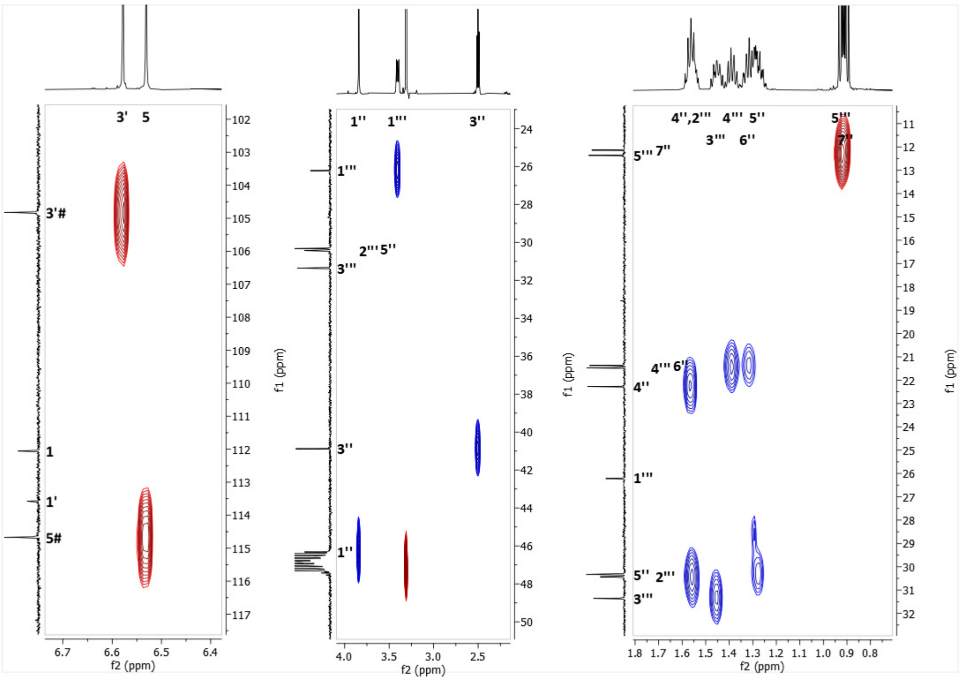

Figure S19. ^{1}H,^{13}C-HSQC NMR spectrum (600 MHz, 150 MHz, CD_3OD-d_4) of 3-hydroxyphysodic acid.

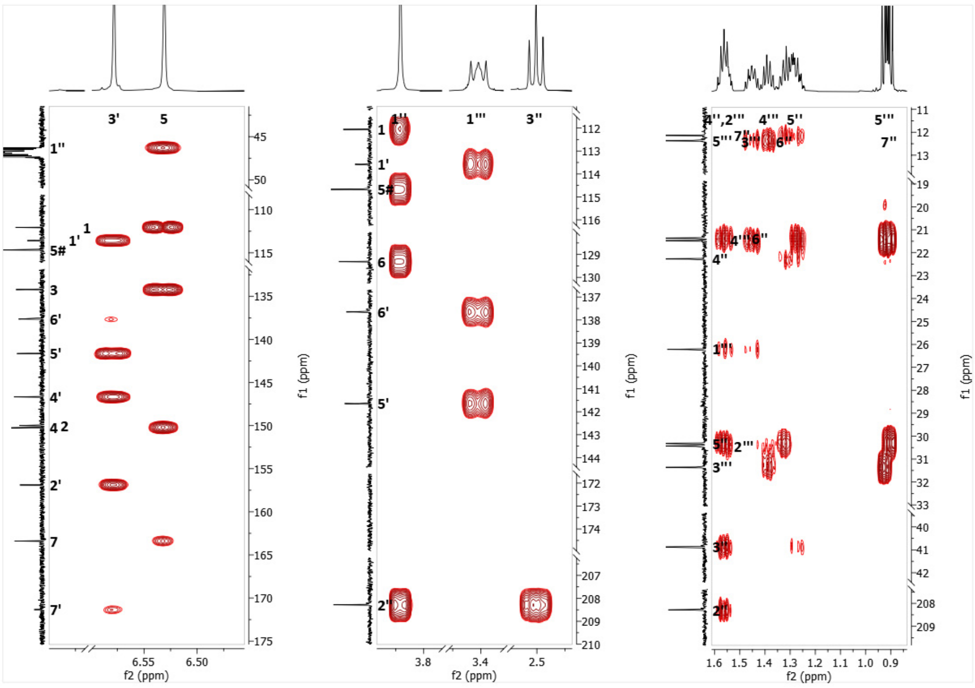

Figure S20. ^{1}H,^{13}C-HMBC NMR spectrum (600 MHz, 150 MHz, CD_3OD-d_4) of 3-hydroxyphysodic acid.

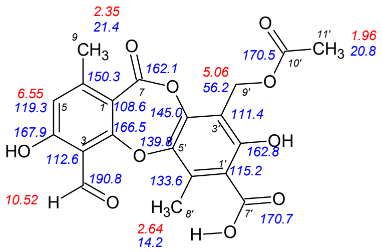

physodalic acid

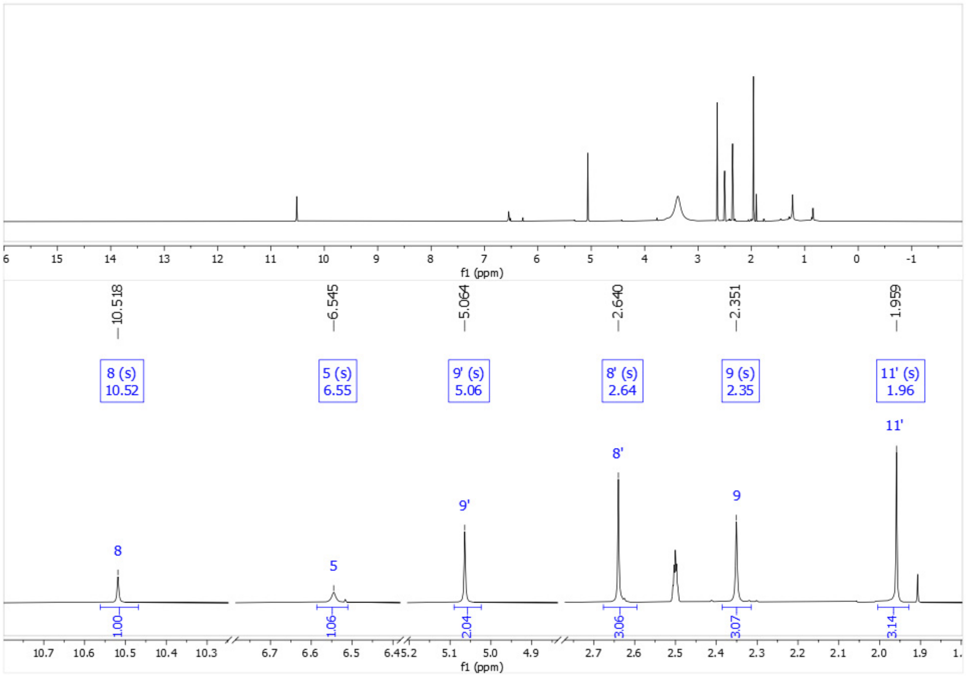

Figure S21. ^{1}H NMR spectrum (600 MHz, DMSO-d$_6$) of physodalic acid.

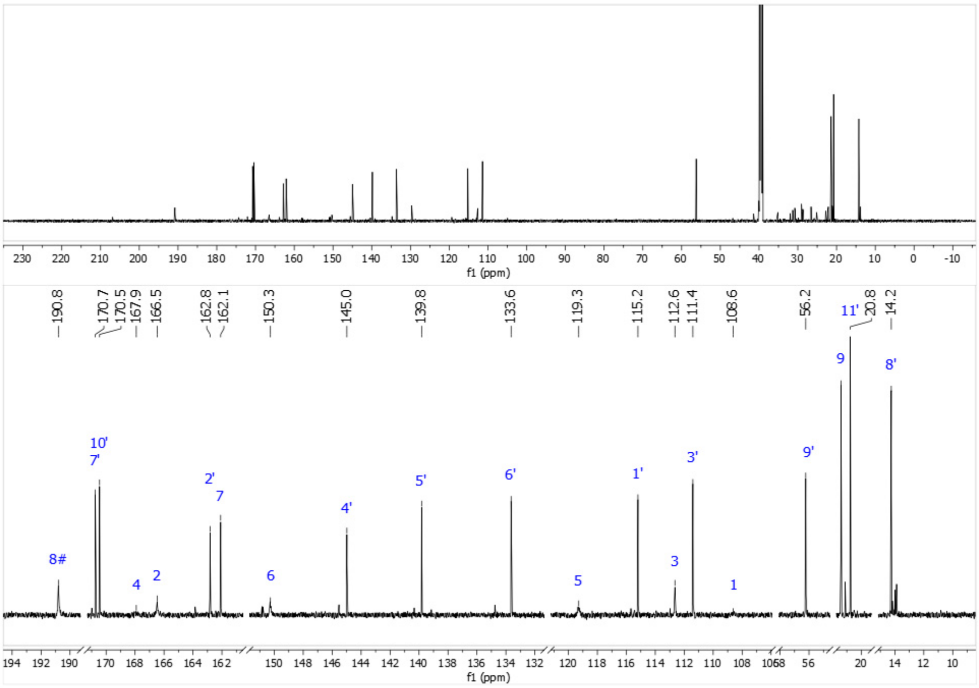

Figure S22. ^{13}C NMR spectrum (150 MHz, DMSO-d$_6$) of physodalic acid.,

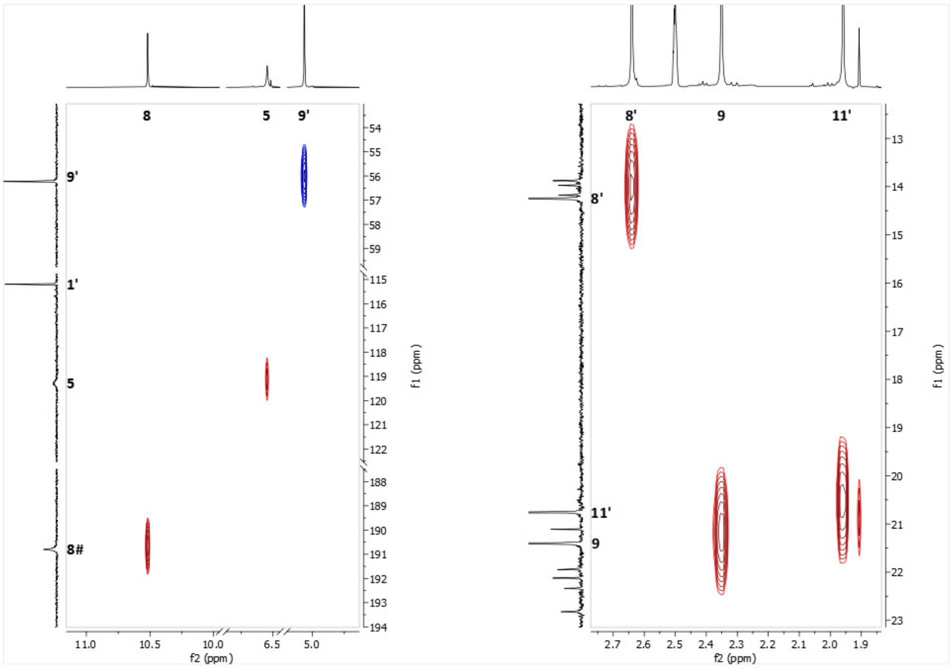

Figure S23. ^{1}H,^{13}C-HSQC NMR spectrum (600 MHz, 150 MHz, DMSO-d$_6$) of physodalic acid.

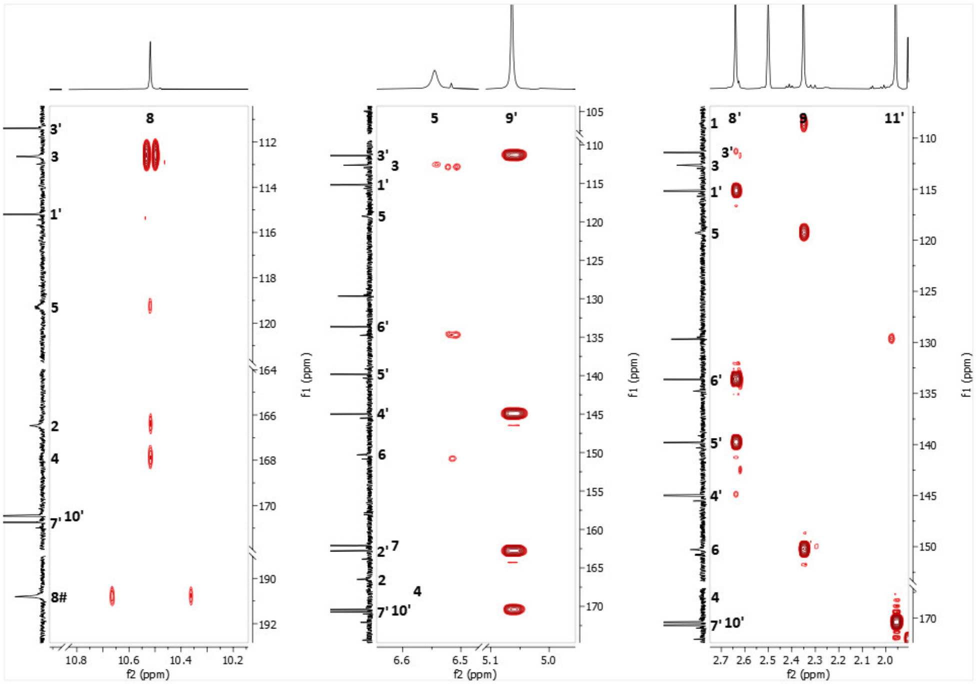

Figure S24. ^{1}H,^{13}C-HMBC NMR spectrum (600 MHz, 150 MHz, DMSO-d$_6$) of physodalic acid.
